# Ultrasonic visualization technique for anatomical and functional analyses of the sciatic nerve in rats

**DOI:** 10.3389/fnins.2023.1187669

**Published:** 2023-06-29

**Authors:** Xiao-Dong Xu, Lei Lin, Yu-Bei Qiu, Bang-Wei Zeng, Ye Chen, Jun-Le Liu, Cai-Hong Ye, Jia-Li Wang, Pei-Chang Liu, Liang-Cheng Zhang

**Affiliations:** ^1^Department of Anesthesiology, Fujian Medical University Union Hospital, Fuzhou, China; ^2^The Graduate School of Fujian Medical University, Fuzhou, China; ^3^School and Hospital of Stomatology, Fujian Medical University, Fuzhou, China; ^4^Administration Department of Nosocomial Infection, Fujian Medical University Union Hospital, Fuzhou, China; ^5^Department of Anesthesiology, Xiamen Third Hospital, Xiamen, China

**Keywords:** ultrasound, anatomy, sciatic nerve, cross-sectional area (CSA), real-time guided, peripheral neuropathy

## Abstract

**Background and objective:**

Ultrasound has been widely used in the diagnosis and minimally invasive treatment of peripheral nerve diseases in the clinic, but there is still a lack of feasibility analysis in rodent models of neurological disease. The purpose of this study was to investigate the changes in the cross-sectional area of the sciatic nerve of different genders and body weights and to explore the effectiveness and reliability of an ultrasound-guided block around the sciatic nerve in living rats.

**Methods:**

Using ultrasound imaging anatomy of the sciatic nerve of rats, the cross-sectional area of the sciatic nerve in rats of different genders from 6 to 10 weeks old was calculated, and then analyzed its correlation with body weight. Further analyses were conducted through behavioral and cadaveric studies to evaluate the feasibility of ultrasound-guided perineural injection of the sciatic nerve in rats.

**Results:**

We first reported that the sciatic nerve cross-sectional area of rats was increased with age (*F* = 89.169, *P* < 0.001), males had a higher sciatic nerve cross-sectional area than females (*F* = 60.770, *P* < 0.001), and there was a positive correlation with body weight (*r*_Male_ = 0.8976, *P* < 0.001; *r*_Female_ = 0.7733, *P* < 0.001). Behavioral observation of rats showed that the lower extremity complete block rate was 80% following the administration of drugs around the sciatic nerve under ultrasound guidance and staining with methylene blue occurred in all sciatic nerves and surrounding muscles and fascia using 20 ultrasound-guided injections.

**Conclusions:**

Ultrasound visualization technology can be used as a new auxiliary evaluation and intervention therapy for animal models of peripheral nerve injury, and will provide overwhelming new references for the basic research of neurological diseases.

## 1. Introduction

With the evolution of medical technology, high-frequency ultrasound has become a widely used imaging tool for the peripheral nervous system. This technique can display the relationship between nerves and surrounding tissues well and is of great value in the diagnosis and evaluation of peripheral nerve diseases, such as nerve entrapment syndrome, hereditary diseases, trauma, and nerve tumors (Hollister et al., 2012). Among the numerous evaluation metrics reported by ultrasound, cross-sectional area (CSA) is the major parameter used to quantify the severity of peripheral neuropathy in clinical practice owing to its good consistency and repeatability both within and across observers (Kara et al., 2012; Tagliafico et al., 2012; Gallardo et al., 2015; Moran et al., 2020). The histology of the peripheral nerves of rats is similar to that of human beings (Mackinnon et al., 1985), so its sciatic nerve is recognized as an ideal model for experimental studies of various peripheral neuropathies (Evans, 2001). In recent years, high-frequency ultrasound has been applied in basic experiments to evaluate the neuropathies of diabetes mellitus and sciatic nerve extrusion in rat models by observing and quantitatively measuring the CSA of the sciatic nerve, the thickness of the nerve, and the echo intensity within the nerve (Huang et al., 2016; Ni et al., 2019). However, there is a lack of systematic analysis of the normative values of rat sciatic nerve CSA measured using ultrasound.

Owing to its advantages of portability, visualization, and no radiation, ultrasound has become an effective tool for precise injury target intervention in the treatment of peripheral nerve diseases (Bomberg et al., 2018). Moreover, ultrasound-guided perineural administration has also been gradually extended to large animal experiments, such as those involving sheep and dogs (Waag et al., 2014; Marolf et al., 2019). However, at present, there are only a few reports on small rodent models. Previously, the administration of drugs to the sciatic nerve in rats was based on anatomical positioning or *in situ* re-incision to expose the nerve (Thalhammer et al., 1995; Brummett et al., 2011), which had shortcomings such as blind injection, inadequate treatment, and secondary trauma to animals. If ultrasound-guided drug delivery technology can be applied to rat models that are commonly utilized in the field of nerve injury research, it will provide more direct scientific evidence for future clinical treatment and enhance the welfare of the experimental animals.

In this experiment, we intended to explore the feasibility of applying ultrasound technology in the study of rat sciatic nerves through ultrasound imaging anatomy, animal behavior, and gross anatomical observation and to provide technical support for other subsequent studies.

## 2. Materials and methods

The ethics committee approval for this study was obtained from the Fujian Medical University Laboratory Animal Management Committee (No. IACUC FJMU 2022-0805) to comply with the Guide for the Care and Use of Laboratory Animals published by the National Institutes of Health.

### 2.1. Animals

A total of seven male and nine female Sprague–Dawley (SD) rats (6 weeks of age, 150–200 g in body weight) were purchased from Shanghai Slaccas Laboratory Animal Co. Ltd., China. All rats, as part of other experimental studies requiring euthanasia, were housed at the Laboratory Animal Center of Fujian Medical University, and a standard diet and water *ad libitum* were provided to the rats in standardized conditions (dark–light cycle, 12/12 h; room temperature, 24 ± 2°C; relative humidity, 60 ± 5%). The rats were acclimatized to the laboratory environment for at least 7 days before the experiment, and a considerable effort was made to reduce the number of rats used and the suffering they endured.

### 2.2. Ultrasound examination and measurement

**Figure 1:**
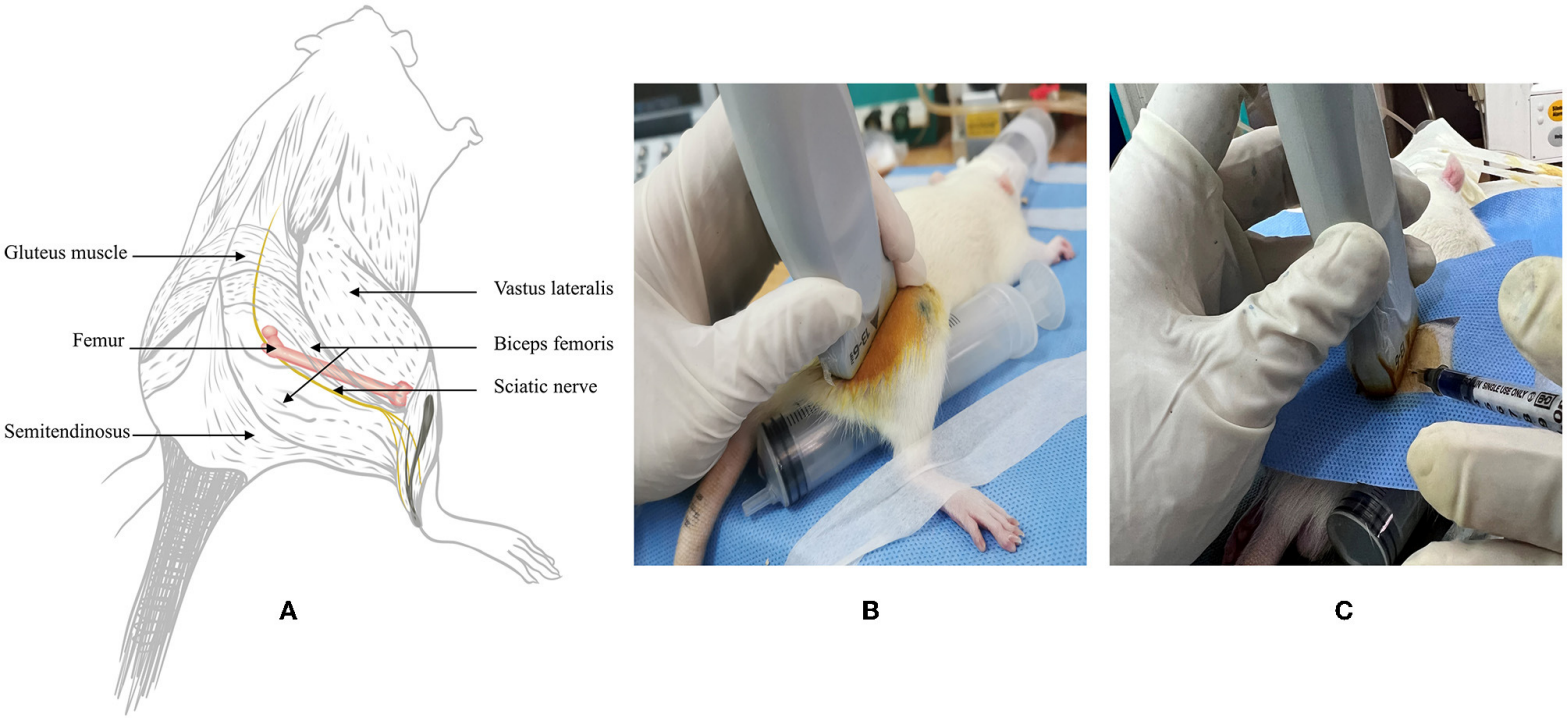
Schematic illustrating anatomy and ultrasound examination in rats. **(A)** Drawing of the rat low limb showing the sciatic nerve, bone, and surrounding muscles. **(B)** Rat‘s position and probe placement during the ultrasound examination. **(C)** 0.5 mm outside the midline of the probe, ultrasound-guided around the sciatic nerve injection in an in-plane approach.

Bilateral sciatic nerve ultrasound imaging and measurement were performed at 6, 7, 8, 9, and 10 weeks after birth by a senior clinical anesthesia-ultrasound subspecialist at a fixed time each week, and the body weight was recorded. All procedures were performed using a color ultrasound system with a sterile ultrasound probe protective sheath (L25 linear probe; musculoskeletal mode, frequencies 6–13 MHZ; Sonosite, USA) under 4–5% sevoflurane (Hengrui Pharmaceuticals Co., Ltd, China) anesthesia (Fraction of inspiration O_2_ 40%, 2 L/min). The SD rats were prone-positioned, the scanning side lower limb was fixed by hip extension, and a 20-ml syringe was placed under the femur to enlarge the contact surface. The relevant gain and depth were adjusted to the rat anatomy to locate the sciatic nerve in the medial femur and deep side of the biceps femoris (Figures 1A, B). To calculate the CSA, the hyperechoic epineurial rims surrounding the sciatic nerve were measured before bifurcation and the area formula was applied (length diameter × short diameter × π ÷ 4), three independent images were intercepted to calculate, and the average value was taken. The contralateral sciatic nerve was examined and measured using the same method.

### 2.3. Ultrasound-guided injection around the sciatic nerve

After the fifth ultrasound measurement, 10 rats (five males and five females) were randomly selected to inject around 10 right or left sciatic nerves. The anesthesia, postural placement, and ultrasound localization of the sciatic nerve procedures were performed as previously described; when the nerve was clearly visible on ultrasound, the 26G puncture needle was inserted at a distance of 5 mm away from the midpoint of the probe using a short-axis in-plane technique (Figure 1C). Once the tip of the needle punctured the gluteal fascia beside the sciatic nerve, there was a slight sense of breakthrough, and the corresponding liquid was injected. Using the same technique, 200 μl of 1% lidocaine (Pujinlinzhou Pharmacy, Shanghai, China) + 0.1% methylene blue (Jumpcan Pharmaceutical, China) was administered adjacent to the sciatic nerve (group L), and 200 μl of 0.1% methylene blue was injected on the contralateral side of each rat (group C). A random number table was used to assign group L to either the right or left sciatic nerve and control group C to the contralateral limb.

### 2.4. Assessment of motor and sensory dysfunction of the lower extremities in rats

Rats were placed on a stainless steel mesh frame before and 15, 30, 45, 60, 75, 90, 120, and 150 min after the peri-sciatic nerve injection, and each limb was tested for motor ability and nociceptive sensation by a blinded investigator using grading scales for motor and sensory hypofunction (Table 1) (Gianolio et al., 2005; Lirk et al., 2012) until all functions returned to normal. A total block was defined as a motor dysfunction score = 2 and a sensory dysfunction score = 3, while a motor dysfunction score = 0 and a sensory dysfunction score = 0 were defined as an invalid block. The other score combinations were defined as an incomplete block. We defined block duration as the time taken for the block to completely disappear (i.e., when the motor and hypoesthesia scores both drop to 0).

**Table 1:** Scoring method for the rat sciatic nerve blockade study.

**A**.	
**Score**	**Observations**
2	Severe block—Rats failed to grab grids when they elevated their hindquarters, dragging their legs
1	Partial block—Rats walked by gathering the forepart of their foot, keeping it sideways, and their ability to grasp grids was limited
0	Normal—Normal walking and grasping abilities were observed in rats
**B**.	
**Score**	**Observations**
3	Complete block—Neither nocifensive behaviors nor vocalization was observed
2	Moderate block—Slow leg withdrawal or flexion accompanied by vocalization
1	Minimal block—Flexing the leg quickly, moving the body sideways, or evading and loudly vocalizing
0	Normal—As shown above, baseline with quick nociceptive responses

The serrated forceps were used to pinch the lateral metatarsal skin fold, increasing the pressure for 2 s manually, and iatrogenic injuries were prevented by not clamping the first gear. Once a positive reaction was observed, the forceps were loosened immediately.

### 2.5. Anatomical study

Following the assessment of sciatic nerve functions, the rats were euthanized under continuous inhalation anesthesia induced by 8% sevoflurane. An incision was made in the lateral thigh of the rat, and the fascial membrane was bluntly separated between the sacral and pelvic heads of the biceps femoris and the pelvic head to expose the sciatic nerve. The distribution of methylene blue in the nerve and surrounding muscle connective tissue was evaluated. The success and reliability of the injection were determined by the presence of the stained perineural connective tissue touching the sciatic nerve directly and/or staining the nerve itself. If there was no direct contact between the stained tissues and the nerve, the perineural injection was considered to have failed.

### 2.6. Statistical analysis

SPSS Statistics 25 (IBM, USA) was used to organize and analyze the obtained data. Counting data were expressed as an example (percentage), while metrological data were tested for normality and expressed as the mean ± standard deviation ($\bar{x}$ ± s) in cases of normal distribution and as the median (interquartile spacing) *M* (*P*_25_, *P*_75_) for those not conforming to normal distribution. The sciatic nerve CSA was compared at different time points using repeated measures analysis of variance (ANOVA), the duration of motor, and sensory block using the Mann–Whitney U-test. The Pearson correlation test was conducted to evaluate the correlation between the sciatic nerve CSA and body weight. A *P-*value of < 0.05 was considered to represent a statistically significant difference. GraphPad Prism 9.0 (GraphPad Software, USA) was used for graphing.

## 3. Results

### 3.1. Ultrasound imaging characteristics and developmental regularity of the sciatic nerve in rats

**Figure 2:**
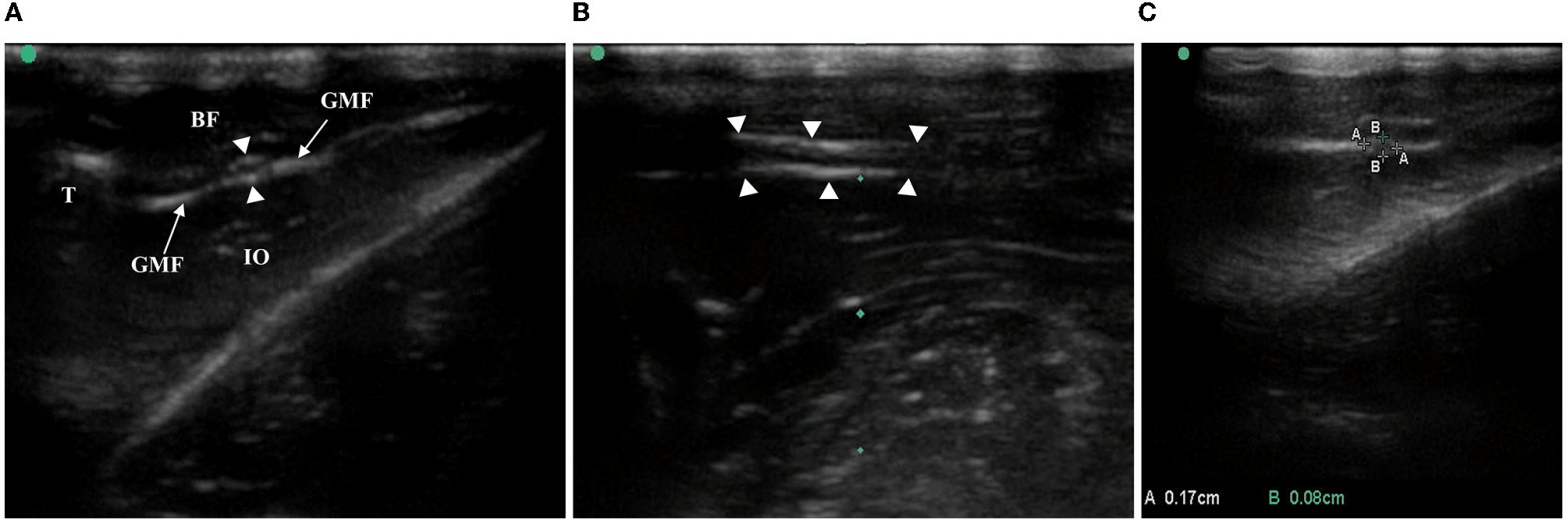
Anatomy and CSA measurement of rat sciatic nerve by ultrasound. **(A)** In the middle/lower femur, the white, highlighted femur cortex and the anechoic region below it, the biceps femoris with hypoecho at the superficial level, the obturator internus with hypoecho at the deep level, and the sciatic nerve surrounded by the gluteal fascia with high echo were observed in the short-axis section of the sciatic nerve of the ultrasound image. T, thighbone, BF, biceps femoris, GMF, gluteus muscle fascia, IO, internal obturator, arrows with triangle-sciatic nerve. **(B)** Long-axis section of the sciatic nerve: arrows with triangle-sciatic nerve. **(C)** Short-axis section of the sciatic nerve, AA is the long diameter of the nerve and BB is the short diameter of the nerve.

The high-frequency ultrasound images showed that the oval hypoechoic sciatic nerve was surrounded by the hyperechoic outer membrane in the short-axis section of the nerve and gradually left the femur from proximal to distal. Above the knee joint, the sciatic nerve bifurcated into the peroneal, tibial, and sural nerves, and the oval structure disappeared (Figure 2A). In the long-axis section of the nerve, the hyperechoic outer nerve membrane surrounding the hypoechoic sciatic nerve bundle was also observed, and the nerve bundle membrane exhibited no obvious echo (Figure 2B).

**Figure 3:**
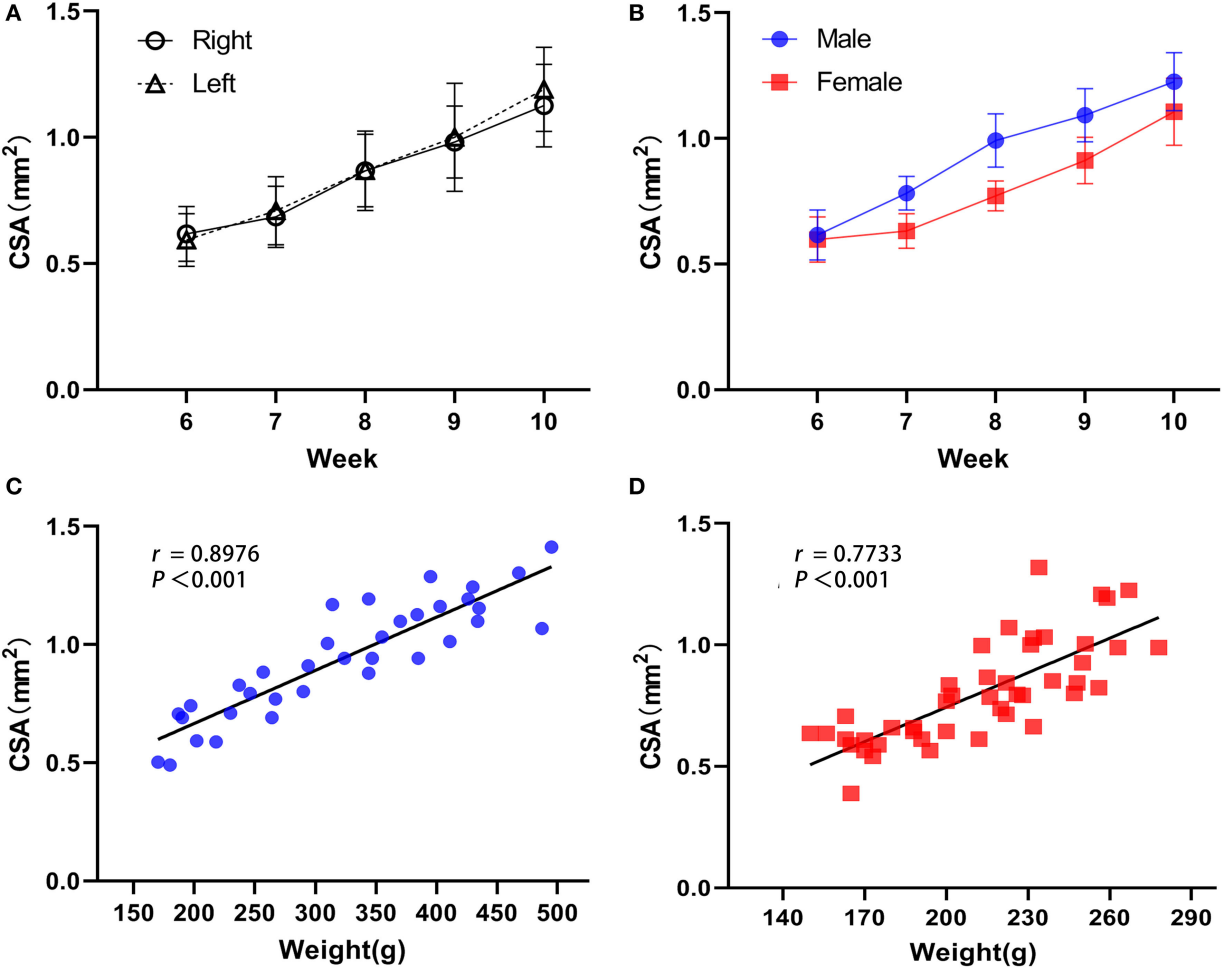
Developmental regulation of the rat sciatic nerve. **(A)** Overall, the difference in sciatic nerve CSA was statistically significant between time points (*F* = 89.169, *P* < 0.001), and the difference between right and left rat sciatic nerve CSA was not statistically significant (*F* = 0.288, *P* = 0.596), while there was no interaction between the right and left CSA and rat week age (*F* = 0.483, *P* = 0.748). **(B)** There was no interaction effect between sciatic nerve CSA and age in rats of different sexes (*F* = 2.301, *P* = 0.069), but the difference in CSA between genders was statistically significant, with males greater than females overall (*F* = 60.770, *P* < 0.001, Figure 3B). **(C)** Sciatic nerve CSA was positively correlated with body weight in male rats. **(D)** Sciatic nerve CSA was positively correlated with body weight in female rats (male *n* = 7 and female *n* = 9, $\bar{x}\pm s$).

The long and short diameters of the intact sciatic nerve before bifurcation were measured along the hyperechoic outer nerve membrane in the short-axis section of the nerve, and the CSA was calculated (Figure 2C). Together, the bilateral sciatic nerve CSA increased gradually from the 6th week to the 10th week after birth, there were statistically significant differences between the time points (*F* = 89.169, *P* < 0.001), the CSA was not statistically significant between right and left limbs (*F* = 0.288, *P* = 0.596), and there were no significant cross-level interactions between the CSA of the bilateral sciatic nerve and age (*F* = 0.483, *P* = 0.748). Thus, the mean value of the left and right CSA was used for follow-up analysis (Figure 3A, Table 2). We found that there was no interaction between the CSA of different sex and age (*F* = 2.301, *P* = 0.069) from the 6th to the 10th week after birth, but the CSA of males was larger than that of the females overall (*F* = 60.770, *P* < 0.001). Moreover, a positive correlation was identified between the CSA of the sciatic nerve and body weight in rats of different genders (*r*_Male_ = 0.8976, *P* < 0.001; *r*_Female_ = 0.7733, *P* < 0.001; Figures 3B–D, Table 3).

**Table 2:** Cross-sectional area of the left and right sciatic nerve in rats of different ages.

		**6W**	**7W**	**8W**	**9W**	**10W**	***F***	***P***
Right	CSA (mm^2^)	0.62 ± 0.11	0.69 ± 0.12	0.87 ± 0.16	0.98 ± 0.14	1.13 ± 0.16	45.881	< 0.001
Left	CSA (mm^2^)	0.59 ± 0.10	0.71 ± 0.13	0.87 ± 0.14	1.00 ± 0.21	1.19 ± 0.17	43.552	< 0.001

*n* = 16, $\bar{x}\;\pm$ s.

**Table 3:** Sciatic nerve CSA and body weight in rats of different ages.

		**6W**	**7W**	**8W**	**9W**	**10W**
Male	CSA (mm^2^)	0.62 ± 0.38	0.78 ± 0.25	0.99 ± 0.40	1.09 ± 0.40	1.23 ± 0.44
	Weight (g)	192.00 ± 15.59	255.86 ± 20.33	329.00 ± 26.03	391.86 ± 35.01	444.14 ± 39.82
Female	CSA (mm^2^)	0.60 ± 0.30	0.63 ± 0.23	0.77 ± 0.20	0.91 ± 0.31	1.11 ± 0.45
	Weight (g)	165.00 ± 8.86	191.11 ± 13.07	220.44 ± 13.15	235.00 ± 16.16	251.89 ± 17.75

Male *n* = 7, Female *n* = 9, $\bar{x}$ ± s.

### 3.2. Ultrasound imaging of injection around the sciatic nerve in rats

**Figure 4:**
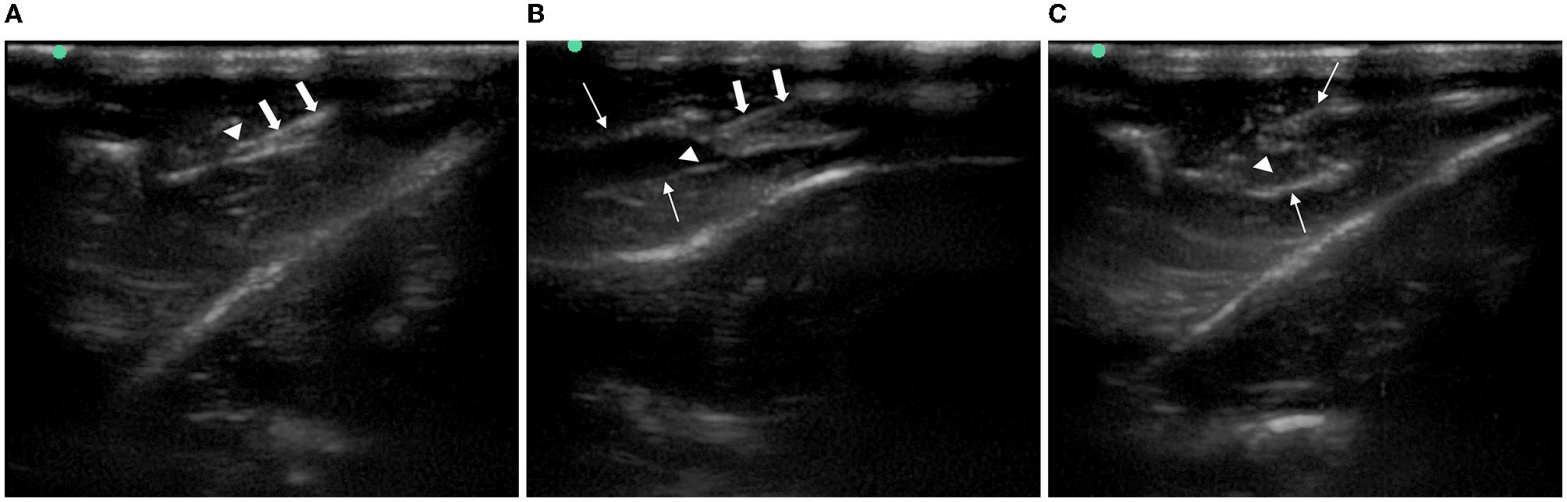
Imaging of ultrasound-guided injection around the rat sciatic nerve. **(A)** Needle reached the gluteal muscle fascia beside the sciatic nerve. **(B)** Superficial and deep layers of gluteal muscle fascia were separated after injection. **(C)** Sciatic nerve was immersed in an anechoic solution. Arrows with triangle: sciatic nerve. Thick arrow: needle. Thin arrow: the gluteal muscle fascia.

The identification of the sciatic nerve just before bifurcation using ultrasound in the short-axis section was successful in all rats; the puncture needle was inserted at 0.5 cm to the midpoint of the side-open probe, and the tip and stem were visible throughout the ultrasonic plane. After puncturing the hip fascia and drawing back the needle tip without blood, we injected the corresponding liquid that could be seen to expand the superficial and deep layers of the hip fascia, and the anechoic fluid was fully wrapped around the sciatic nerve (Figure 4).

### 3.3. The behavioral observation of rats after the ultrasound-guided sciatic nerve blockade

**Figure 5:**
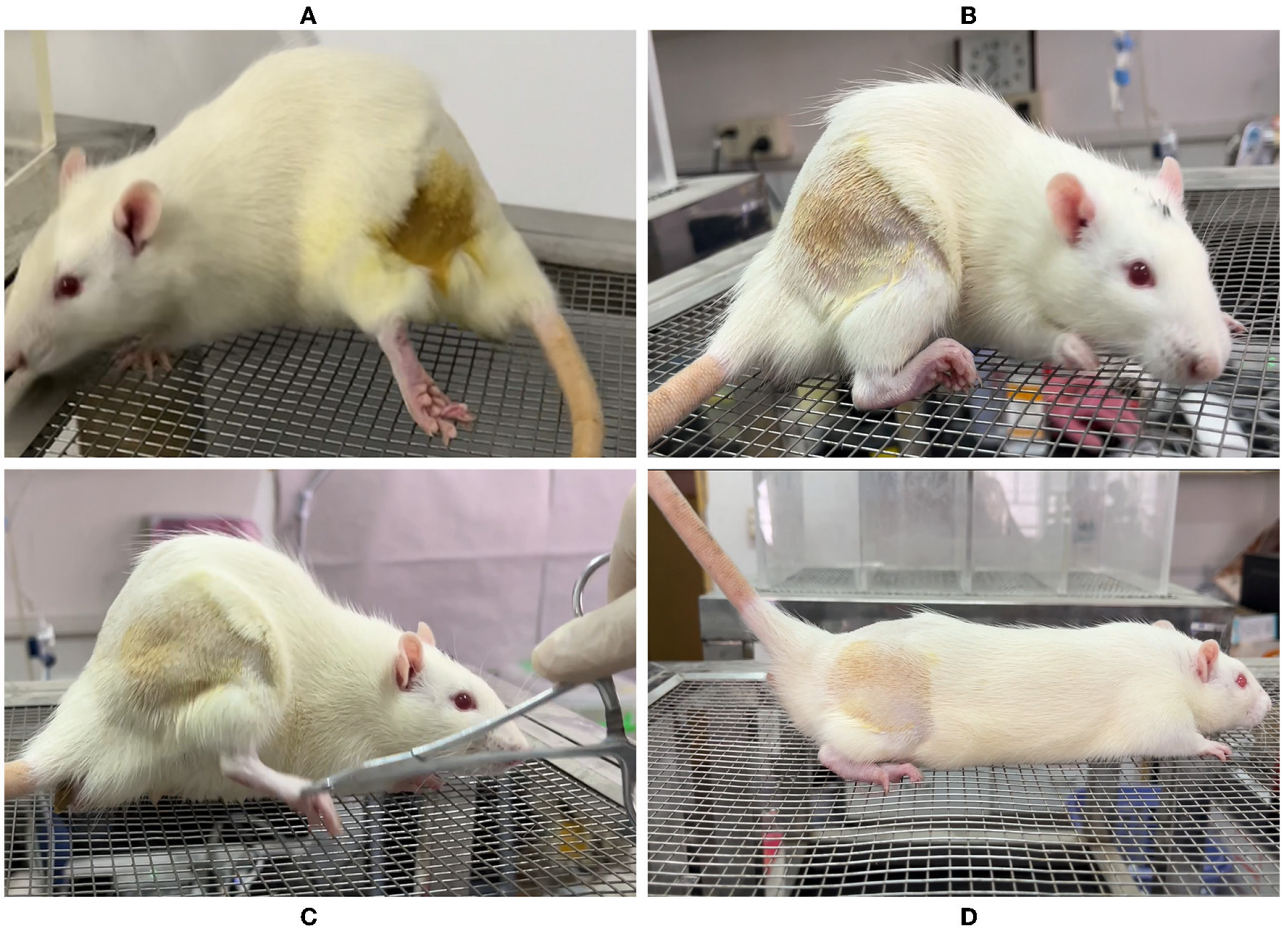
Behavioral observation of rats after ultrasound-guided injection of lidocaine around the sciatic nerve. **(A)** Foot dragging on the side of lidocaine injection. **(B)** Toe flexion, foot valgus, and grasping grid weakness were observed on the side of lidocaine injection. **(C)** Clamp the lateral skin of the foot on the saline injection side, and the rats shouted loudly with rapid retraction of the lower limbs. **(D)** Grasping grid with toes was normal on the saline injection side.

All the rats received an ultrasound-guided injection to the bilateral sciatic nerve. A total of seven rats were injected with 200 μl 1% lidocaine + 0.1% methylene blue into the right side and three rats were injected into the left side. In group L, the lower extremity of the injection side showed movement disorder after successful injection, exhibited by foot-dragging and valgus, curling of toes, and inability to grab the grids upon elevation of the hindquarters, while the complete sensory block showed no obvious response or vocalization when the skin folds on the lateral metatarsal of the foot was pinched with a serrated forceps. On the contrary, in group C, the lower extremity motor and sensory function of the injected side was normal after injection (Figure 5). No other adverse reactions were observed during and after perineural injection.

### 3.4. Motor and sensory impairment scores in rats

**Figure 6:**
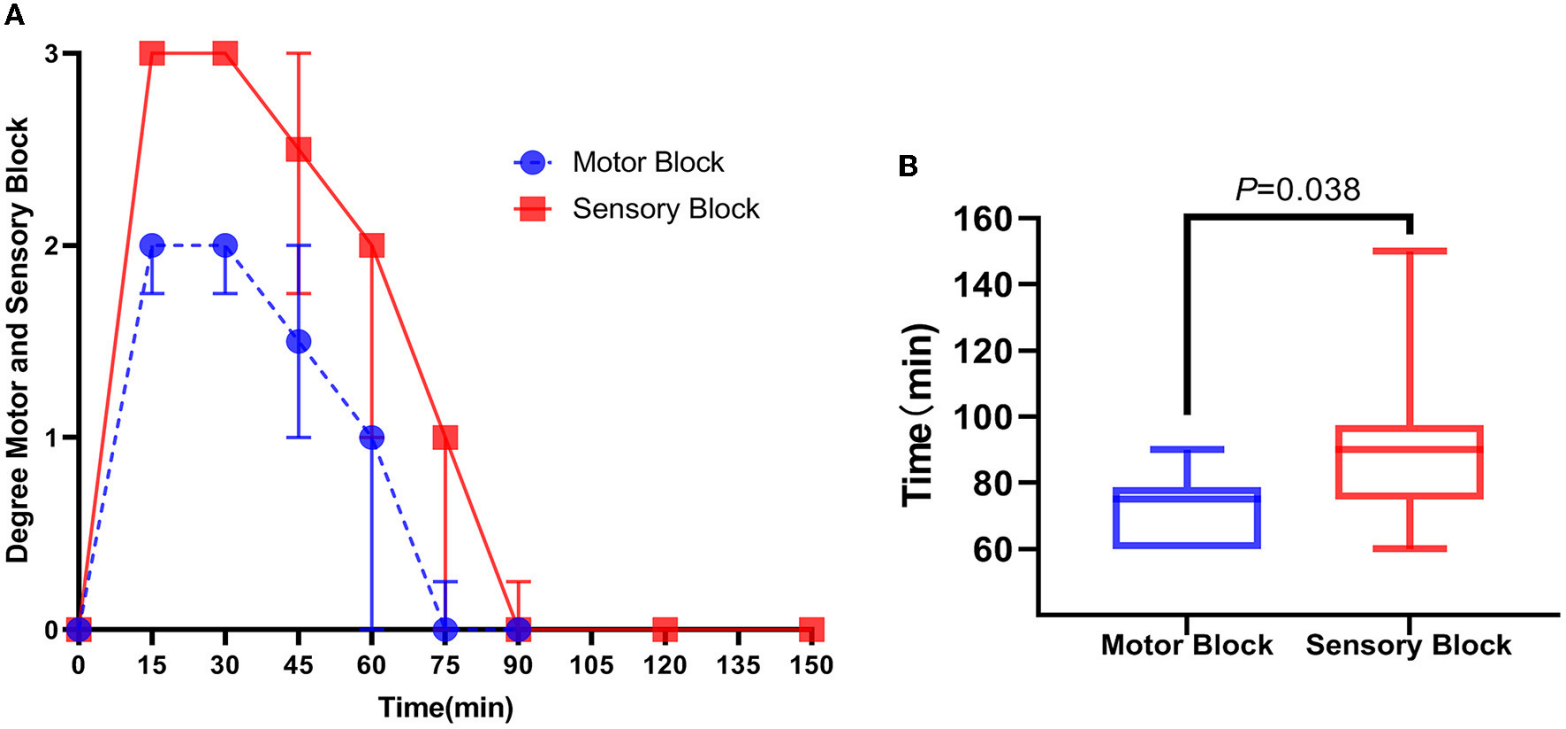
Score of motor and sensory dysfunction in rats after ultrasound-guided injection of lidocaine around the sciatic nerve. **(A)** Within 150 min after lidocaine injection, the time trend of the score of motor and sensory dysfunction of rats was expressed as the median (interquartile distance). **(B)** Duration of motor block in rats after lidocaine injection was smaller than that of sensory block, expressed as median (interquartile distance) and passed the Mann–Whitney *U*-test, *P* = 0.038, *n* = 10.

There was no difference in the lower extremity behavioral test between groups L and C before injection; all rats had a hypokinesia and hypoesthesia score of 0. In group L, all lower limbs were blocked to different degrees: eight cases (80%) were completely blocked (hypokinesia score = 2 and hypoesthesia score = 2), nine cases (90%) were completely blocked in sensory function (hypoesthesia score = 3), and eight cases (80%) were severely blocked in motor function (hypokinesia score = 2). Group C displayed an ineffective block (hypokinesia score = 0 and hypoesthesia score = 0). A significant difference was found between the duration of the motor block [75 (60,79) min] and sensory block [90 (75,98) min] (*P* = 0.038, Figure 6). Motor and sensory functions of all rats were restored to normal levels within 150 min after injection.

### 3.5. Cadaver study

**Figure 7:**
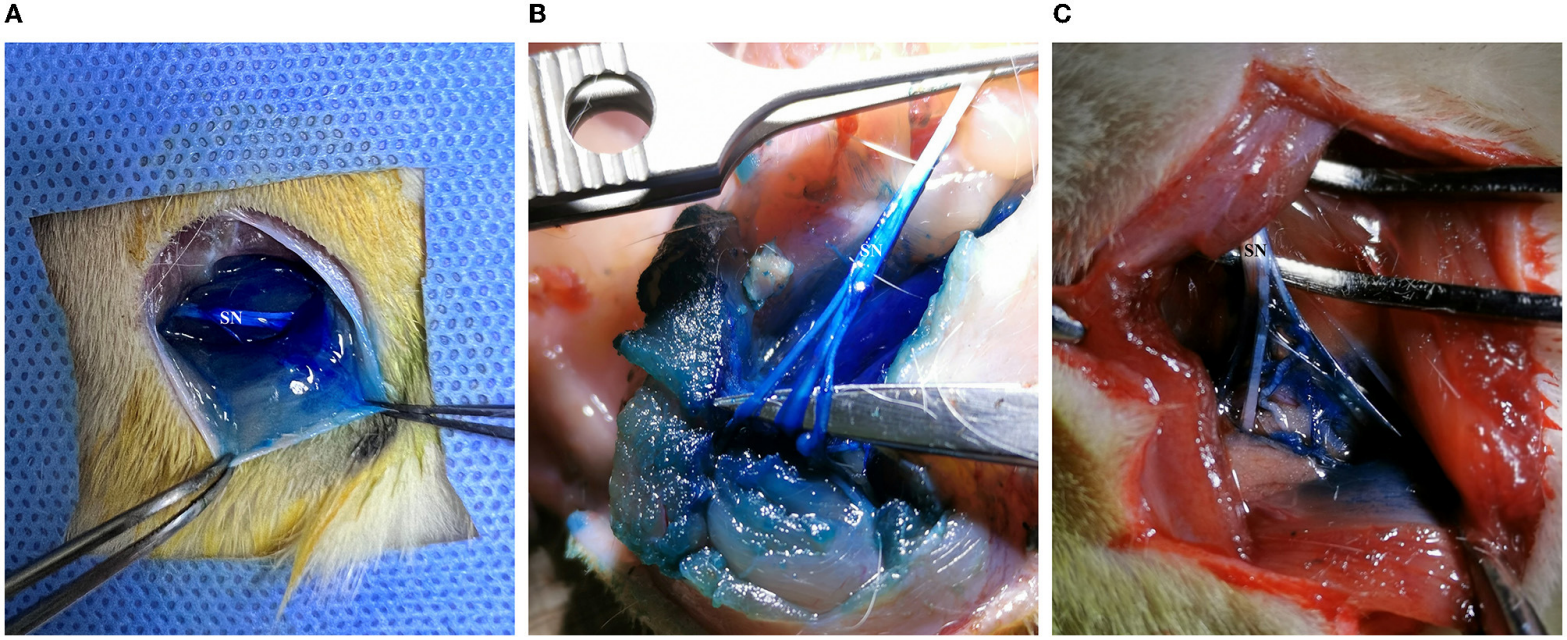
Methylene blue staining of the sciatic nerve and surrounding tissue in rats. **(A)** Methylene blue solution-infiltrated sciatic nerve trunk, surrounding muscles, and soft tissue. **(B)** The trunk and branches of the sciatic nerve, the surrounding muscle, and soft tissue were fully stained. **(C)** The coverage of methylene blue staining in the main stem and branches of the sciatic nerve was inadequate.

In 20 cases of ultrasound-guided peri-sciatic nerve injections, all sciatic nerve, perineural muscle, and connective tissues were successfully stained by methylene blue staining (100% accuracy) (Figures 7A, B). However, in two cases in group L, incomplete coverage of methylene blue staining was found when tracing the sciatic nerve branching into the sural, peroneal, and tibial nerve, and the staining coverage of the sciatic nerve trunk was poor (Figure 7C).

## 4. Discussion

The present study involved seven male and nine female SD rats who were born to the same mother at the same time, which ensured the consistency of the growth and development of the subjects. The CSA of nerves is a commonly used ultrasonic measurement index in the study of peripheral neuropathies. Previous studies have confirmed that CSA can quantitatively reflect the severity of peripheral neuropathy, especially in nerve entrapment neuropathy with impaired blood return and cell edema, which can lead to decreased nerval echo and an enlarged nerval CSA, and in infectious lesions, which exhibit increased intraneural vascularization and CSA (Kara et al., 2012). Several classical peripheral nerve injury models, such as chronic constriction injury (CCI) and partial sciatic nerve ligation (PSNL), all interfered with the sciatic nerve trunk in the mid-femur, which can result in a diffuse swelling at the site of sciatic nerve ligation, as well as both proximal and distal to the ligation site (Bennett and Xie, 1988; Seltzer et al., 1990; Pacini et al., 2010; Di Cesare Mannelli et al., 2013). Ni et al. (2019) described the dynamic changes of ultrasound images of the rat sciatic nerve in a crush injury model and found an increase in nerve thickness, a decrease in the internal echo signal, and blurred and rough images of the outer nerve membrane after injury. These findings further confirm the auxiliary evaluation effect of high-frequency ultrasound in traditional animal peripheral nerve injury models. However, the measurement was limited to the thickness of the injured nerve in the long-axis views, with no analysis of the nerve CSA. Although the CSA of the sciatic nerve may vary between different locations (Huang, 2016), measurements of the CSA in the short-axis view have been confirmed as consistent and dependable, and ultrasound imaging techniques are simpler and easier to promote. As such, in this study, the location near the bifurcation point of the sciatic nerve was uniformly selected as the CSA measurement target to provide relevant foundational data for the homogenized analysis of subsequent related nerve injury models.

In addition, CSA can be measured using the indirect formula method (long diameter × short diameter × π ÷ 4) or the direct trajectory method, both of which have good consistency and repeatability. However, considering the small size of the rat sciatic nerve and the limitations of ultrasonic equipment in this study, precise CSA could not be directly measured by the trajectory method, so we used the indirect area formula method. Following anaesthetization, the sciatic nerve and surrounding muscles and fascia were clearly displayed by ultrasound imaging, which is consistent with the description provided by Hughey et al. (2022). We found that the CSA of the bilateral sciatic nerves increased gradually from the 6th to the 10th week of life in rats of both genders, and the left and right sides did not differ statistically, which is consistent with previous clinical human ultrasound anatomical studies (Cartwright et al., 2008). Due to the difference in the developmental law of body weight between rats of different genders, the sciatic nerve CSA of male rats was larger than that of females with increasing age but slightly smaller than the results reported by Yun-xia et al. (2013) and Liu et al. (2022). This could be related to where the sciatic nerve was measured and the ages of the rats in different studies. In addition, we also found that the sciatic nerve CSA was highly positively correlated with body weight in rats of different genders, and the correlation coefficients were higher than those between body weight and sciatic nerve CSA in humans reported by Cartwright et al. (2008), further suggesting that the sciatic nerve CSA of rats is less disturbed by other factors and can be used as a reliable indicator for neurological assessment in peripheral nerve injury models.

Currently, ultrasound-guided perineural injection techniques have been gradually applied in preclinical animal studies but mainly in large mammals (Marolf et al., 2019; Lee et al., 2020; Micieli et al., 2021). Meanwhile, for peripheral nerve administration in small rodents such as rats, the vast majority are still based on anatomical landmarks (Thalhammer et al., 1995), incisional direct vision (Brummett et al., 2011), etc. Due to the small injection dose and the blindness of puncture, the anatomical signal-based approach has the potential for inadequate administration. On the other hand, although percutaneous incision is more accurate for injection under direct vision, this procedure can lead to various traumas, such as skin laceration and muscle stripping, and animals may suffer from postoperative pain, hyperalgesia, and related behavioral changes, which in turn may affect subsequent behavioral observations, including the mechanical withdrawal threshold (PMWT) and withdrawal latency (TWL) tests. Hughey et al. demonstrated for the first time the feasibility of ultrasound-guided injection around the sciatic nerve in rats using the methylene blue tracer on corpses, and the presence of methylene blue staining was found in all 40 sciatic nerves with 100% reliability, which provided the basis for our study (Hughey et al., 2022). However, the frozen cadavers used lacked the contraction and extrusion of the perineural muscles, and the diffusion range of local drug liquid was limited, while at the same time, real-time post-injection functional response assessments could not be performed. Lyu et al. placed the radiofrequency needle near the affected sciatic nerve trunk of the spared nerve injury (SNI) rat model accurately with ultrasound guidance and relieved the depression caused by pain through pulse radiofrequency modulation, suggesting that ultrasonic guidance has certain technical advantages in preclinical studies of small animals; however, the reliability of this technique has not been fully demonstrated (Lyu et al., 2023). Therefore, in this part of the experiment, a mixture of methylene blue/lidocaine was used to wrap the drug around the sciatic nerve of live rats under direct ultrasound, comparing the effects of the injection of 1% lidocaine and saline by motor and sensory hypokinesis scores in rats. We found that the blocking efficiency was 100%, including a complete blocking rate of 80%, and the duration of motor and sensory blocking was 75 (60,79) and 90 (75,98) min, respectively, both slightly longer than the blocking times reported by Sasaki et al. (2017), which may be related to the different intervention methods. Although Sasaki et al. (2017) also injected the same dose (1% lidocaine), it was operated under direct visualization of the sciatic nerve with an incision of the skin muscle, and there was a possibility of extravasation of the drug. However, in the present experiment, the local anesthetic solution was more adequately wrapped around the sciatic nerve by a more minimally invasive ultrasound-guided percutaneous injection and remained in the myofascial space for a longer period of time, resulting in a more adequate effect. Next, we revealed by anatomical observation that the sciatic nerve, perineural muscles, and connective tissues of all subjects were successfully stained with methylene blue staining, which is consistent with the reports of Hughey et al. in the cadaver study (Hughey et al., 2022). The above results fully demonstrate that ultrasound-guided administration of rat sciatic nerve is an effective and reliable novel intervention for application in animal studies.

The overall sensory block time of rats in this study was significantly longer than the motor block time, which is related to the different degrees of the block of different nerve fibers, such as Aα, Aβ, and C within the sciatic nerve by lidocaine. In terms of individuals, one case in group L had a partial motor block and one case had an incomplete motor and sensory block. After a gross autopsy, it was found that the coverage of the sciatic nerve trunk and branches of the lower extremity in these two rats were inadequately covered by methylene blue staining, and the degree of the functional block was related to the extent and length of local anesthetic infiltration of the nerve, in accordance with the previously reported view of Raymond et al. (1989). Although ultrasound-guided peripheral nerve injection has the advantages of visualization, effectiveness, and rapidity, there is still a risk of injury to the nerve, surrounding tissues, and vascular structures (Sites et al., 2012). Considering the smaller size of the rat sciatic nerve, there may be some operational difficulties, so the fixed operator in our study was a senior clinical anesthesia-ultrasound subspecialist who was trained in ultrasonic scanning of rat sciatic nerves in the early stage. At the same time, the in-plane technique of the short-axis of the nerve was used during puncture to visualize the entire process of needle entry, and the needle tip was clearly visible next to the target, minimizing the risks of intraneural injection and ensuring study quality and operational consistency. Nevertheless, there were still a few procedures that did not achieve complete functional blockade, which might be related to the low frequency of the ultrasonic probe used in the study or the lack of clear imaging. In the future, more high-frequency ultrasound probes that are specific for use in small animals will be used (Liu et al., 2022). In addition, how to further popularize this technique also deserves attention; we intend to recruit more researchers without ultrasound experience to analyze the learning curve and develop a unified, standardized, and simple protocol to assess the ease of application and generalizability of the ultrasound-guided peri-sciatic nerve injection technique in basic animal studies.

In this experiment, the precise anatomical structure of the sciatic nerve in rats was described using ultrasound, which was simple and rapid, and clear, accurate ultrasound images of the nerve could be obtained. Since male rats were used in most previous studies on peripheral nerve injury models, the relevant ultrasound imaging data obtained were also limited to male rats. Our study included female rats for the first time, systematically reporting on the anatomical baseline value of the sciatic nerve CSA in rats of different genders, and its CSA correlation with weight development, which can provide more comprehensive basic reference data for basic research on nerve injury. On the other hand, we confirmed that the ultrasound-guided precision peri-sciatic nerve injection technique is a reliable method; since there is no need for a skin incision or muscle separation, the surgical trauma of the animal models requiring peri-nerve injection can be greatly reduced. At the same time, for the neuropathic pain models requiring pain assessment and measurement, this technique can also avoid the interference caused by a conventional incision, such as nociceptive hypersensitivity, and could represent a novel approach for repeated drug administration studies. In addition, because of the visualization of the whole process of injection and the clear diffusion of the drug between the peripheral nerve fascia, it is possible that the same therapeutic effect can be achieved with a lower volume of the drug solution, which can further reduce the toxic and side effects of drugs.

## 5. Conclusion

The ultrasound visualization technique is a valuable tool for assessing nerve injury and regeneration, as well as for administering drug injections in the field of neuropathic pain treatment and research. This minimally invasive technique is complementary and represents an improvement over traditional methods. It has the potential to reduce repeated trauma to animals, ultimately improving their welfare. Moreover, this technique can also avoid measurement errors encountered in behavioral tests, such as PMWT in animal models of neuropathic pain under surgical trauma-induced pain. However, compared with clinical practice, this new technology is more challenging to implement in small animal experiments due to limitations such as ultrasound equipment and the small size of the target. Despite these technical challenges, efforts will be made to refine and standardize the relevant operational procedures (including training frequency and optimization of equipment) to ensure the widespread adoption of this technology for the diagnosis and treatment of peripheral nerve diseases in rats in basic research. By enhancing the reliability and precision of the experimental results, the ultrasonic visualization technique can provide better insights into neuropathic pain mechanisms, paving the way for the development of improved treatments.

## Data availability statement

The datasets presented in this study can be found in online repositories. The names of the repository/repositories and accession number(s) can be found below: https://doi.org/10.6084/m9.figshare.22186750.

## Ethics statement

The animal study was reviewed and approved by the Laboratory Animal Management Committee of Fujian Medical University, China.

## Author contributions

X-DX, LL, and Y-BQ: performed the research, collected and assembled the data, and wrote the manuscript. B-WZ, YC, and J-LL: collected and assembled the data and performed the data analysis. C-HY and J-LW: performed the research. L-CZ and P-CL: contributed to the conception and design, revision, and final approval of the manuscript. All authors have read and approved the final manuscript.
